# A novel heterozygous WFS1 variant of uncertain significance in a patient with early-onset diabetes: a case report

**DOI:** 10.3389/fendo.2025.1743282

**Published:** 2026-01-14

**Authors:** Wen Kan, Yunyang Wang, Yu Xue, Xiaoying Zhang, Lili Xu

**Affiliations:** Department of Endocrinology and Metabolic Diseases, Affiliated Hospital of Qingdao University, Qingdao, China

**Keywords:** case report, early-onset diabetes, heterozygote, variant of uncertain significance (VUS), WFS1 gene

## Abstract

**Objective:**

To describe the clinical presentation of a patient with early-onset diabetes and to report a novel heterozygous *WFS1* variant of uncertain significance (VUS) identified in this case. This report aims to contribute to the phenotypic and genotypic spectrum of *WFS1*-related disorders and to discuss the challenges of interpreting VUS in complex clinical scenarios.

**Methods:**

Clinical data were collected from the proband and his family members. Whole-exome sequencing was performed on the proband. Sanger sequencing was subsequently utilized to validate the identified variant in the proband and his parents. A review of the relevant literature was also conducted.

**Results:**

A previously unreported heterozygous missense variant in the WFS1 gene, c.1550G>C (p.Arg517Pro), was identified in the proband. Segregation analysis confirmed that this variant was inherited from his father, a non-diabetic carrier; the mother did not carry the variant. The proband’s clinical phenotype was primarily characterized by early-onset diabetes and its vascular complications. No discernible neurosensory features typical of classical Wolfram syndrome—such as optic atrophy, deafness, or diabetes insipidus—were observed. Following the American College of Medical Genetics and Genomics (ACMG) guidelines, this variant was classified as one of uncertain significance (VUS). The classification was based on the following supporting criteria: PM2_Supporting (due to its extremely low allele frequency of 0.000077 in population databases) and PP3_Moderate (based on in silico predictions from the REVEL tool, which suggested a deleterious effect).

**Conclusion:**

This case report describes a novel WFS1 missense variant of uncertain significance (p.Arg517Pro) identified in a patient with early-onset diabetes. This finding contributes to the growing catalog of rare WFS1 variants and highlights the interpretive challenges they pose. It suggests that WFS1 could be considered in the genetic evaluation of selected cases of early-onset diabetes, even in the absence of full syndromic features. Prospective monitoring of asymptomatic carriers of similar variants may be warranted, pending further evidence to clarify their clinical significance.

## 1 Introduction

Wolfram syndrome (WS) is a rare neurodegenerative disorder characterized by a constellation of features including diabetes mellitus (DM), optic atrophy (OA), deafness (D), diabetes insipidus (DI), and various psychiatric manifestations (1). The syndrome is primarily categorized into two types: WS1 and WS2, caused by pathogenic variants in the WFS1 and CISD2 genes, respectively. WS1 represents the most prevalent form, accounting for approximately 90% of all WS cases (2). The vast majority of WS1 patients harbor pathogenic variants in the WFS1 gene, which encodes the wolframin protein.

Wolframin plays a key role in maintaining proper function of the endoplasmic reticulum (ER), where it regulates crucial processes such as calcium homeostasis, protein folding, and cellular trafficking (3, 4). Mutations in WFS1 result in loss-of-function mutations, leading to a deficiency or functional impairment of wolframin. This disruption in wolframin function hinders calcium transfer between the ER and mitochondria, which exacerbates ER stress and leads to a cascade of protein misfolding. This process triggers cellular dysfunction, which is especially harmful to tissues with high wolframin expression, such as pancreatic β-cells, neurons, and retinal cells. The accumulation of misfolded proteins and the resulting ER stress ultimately leads to progressive organ damage (1, 4). The progressive degeneration of organs affected by Wolfram syndrome results in a range of debilitating symptoms, including diabetes mellitus, optic atrophy (vision loss), sensorineural deafness, and diabetes insipidus, among others. Over time, the syndrome leads to multisystem involvement, including the nervous system, endocrine system, and sensory organs (5).

However, growing clinical and genetic evidence supports the concept of a “Wolfram syndrome spectrum disorder” or “WFS1-related disorder.” Phenotypes can range from the severe, classic syndromic presentation to milder, non-syndromic forms, such as isolated early-onset diabetes or specific neurosensory deficits. While biallelic pathogenic variants are responsible for classic WS, the clinical significance of heterozygous WFS1 variants remains an area of active investigation and debate. The rarity of the disease and the limited number of reported cases for each specific WFS1 variant pose significant challenges for medical geneticists in definitively classifying variants as pathogenic or likely pathogenic (2).

This phenotypic and genotypic heterogeneity poses significant challenges for variant interpretation. Many rare WFS1 variants are classified as VUS, as definitive evidence for pathogenicity is often lacking. Regarding therapeutic options, the use of GLP-1 receptor agonists as a treatment for hyperglycemia in this case appears theoretically reasonable (6). These drugs possess glucose-dependent insulinotropic properties, effectively controlling blood glucose while carrying a relatively low risk of hypoglycemia. Their weight-reducing effects also help improve insulin resistance (7). Additionally, GLP-1 receptor agonists alleviate endoplasmic reticulum stress-induced β-cell death and reduce pancreatic stress, potentially delaying disease progression (8–10). More importantly, preclinical studies have demonstrated β-cell protection and neuroprotective potential, which are highly relevant to the pathophysiology of Wolfram syndrome, suggesting possible benefits beyond glucose control (6, 11). Initial treatment should begin with a low dose, with close monitoring of gastrointestinal tolerance and blood glucose levels.

Herein, we report a patient with a phenotype centered on early-onset diabetes and its complications, who was found to carry a novel heterozygous WFS1 missense variant classified as a VUS. This case highlights the diagnostic and interpretive complexities at the borders of the Wolfram syndrome spectrum and contributes to the understanding of the phenotypic associations of monoallelic WFS1 variants.

## 2 Case presentation

### 2.1 Clinical history

A 28-year-old male visited our endocrinology outpatient clinic on November 19, 2023, with the primary complaint of “elevated blood glucose for more than 9 months.” The patient’s condition was first identified nine months prior during a routine health check, which revealed elevated fasting blood glucose (11–12 mmol/L), urinary ketones (2+), and classic hyperglycemic symptoms including polydipsia, polyuria, and dry mouth. He was diagnosed with diabetes mellitus and began treatment with traditional Chinese medicine, although the specific medications were unclear, and his blood glucose control remained inadequate. Subsequently, the patient sought further diagnosis and treatment at our hospital for comprehensive evaluation and management.

### 2.2 Physical examination

On admission, the patient was afebrile (36.5 °C) with a respiratory rate of 20 breaths per minute, a pulse of 100 beats per minute, and a blood pressure of 138/69 mmHg. Anthropometric assessment revealed a height of 178 cm, weight of 75 kg, body mass index (BMI) of 23.7 kg/m². A comprehensive physical examination, including evaluation of the cardiovascular, respiratory, abdominal, and neurological systems, revealed no significant abnormalities.

### 2.3 Diagnosis and treatment

**Figure 1:**
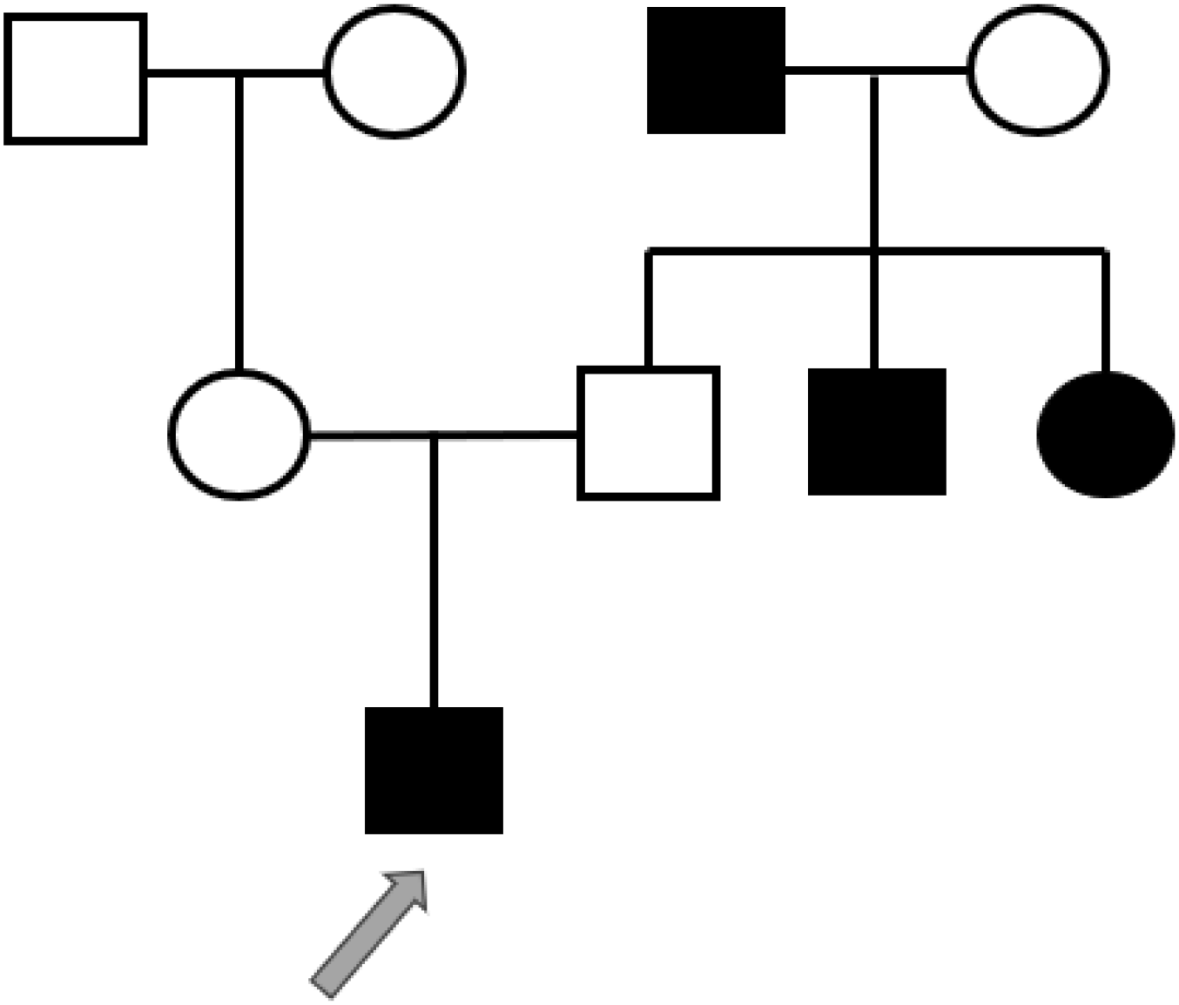
Proband’s family tree: squares represent males, and circles represent females. The black symbol represents individuals with diabetes; the blank symbol represents normal individuals. The arrow indicates the proband.

The patient’s initial evaluation at our hospital revealed a HbA1c of 9.3%, fasting C-peptide of 1.16 ng/mL, and negative insulin autoantibodies. Based on the patient’s family history of diabetes mellitus (involving his paternal grandfather, paternal aunt, and paternal uncle), he was initially diagnosed with diabetes. Given the preserved pancreatic beta-cell function indicated by the C-peptide level, a twice-daily NovoNorm was initiated, resulting in significant glycemic improvement and eventual achievement of HbA1c targets. (**Figure 1**).

In February 2024, the patient developed acute blurred vision and was diagnosed with diabetic retinopathy complicated by vitreous hemorrhage at a local institution. He received hemostatic agents and pan-retinal photocoagulation, with residual visual impairment persisting post-treatment. During subsequent follow-up at our clinic, the insulin regimen was intensified with the addition of metformin and siglitazone sodium. The latter, an insulin sensitizer, contributed to improved insulin resistance and greater glycemic stability in this patient with established retinopathy.

However, the clinical presentation—characterized by young-onset diabetes, suboptimal beta-cell function, and a history of ketonuria—raised strong suspicion for monogenic diabetes. Genetic testing was therefore recommended.

### 2.4 Genetic analysis

**Figure 2:**
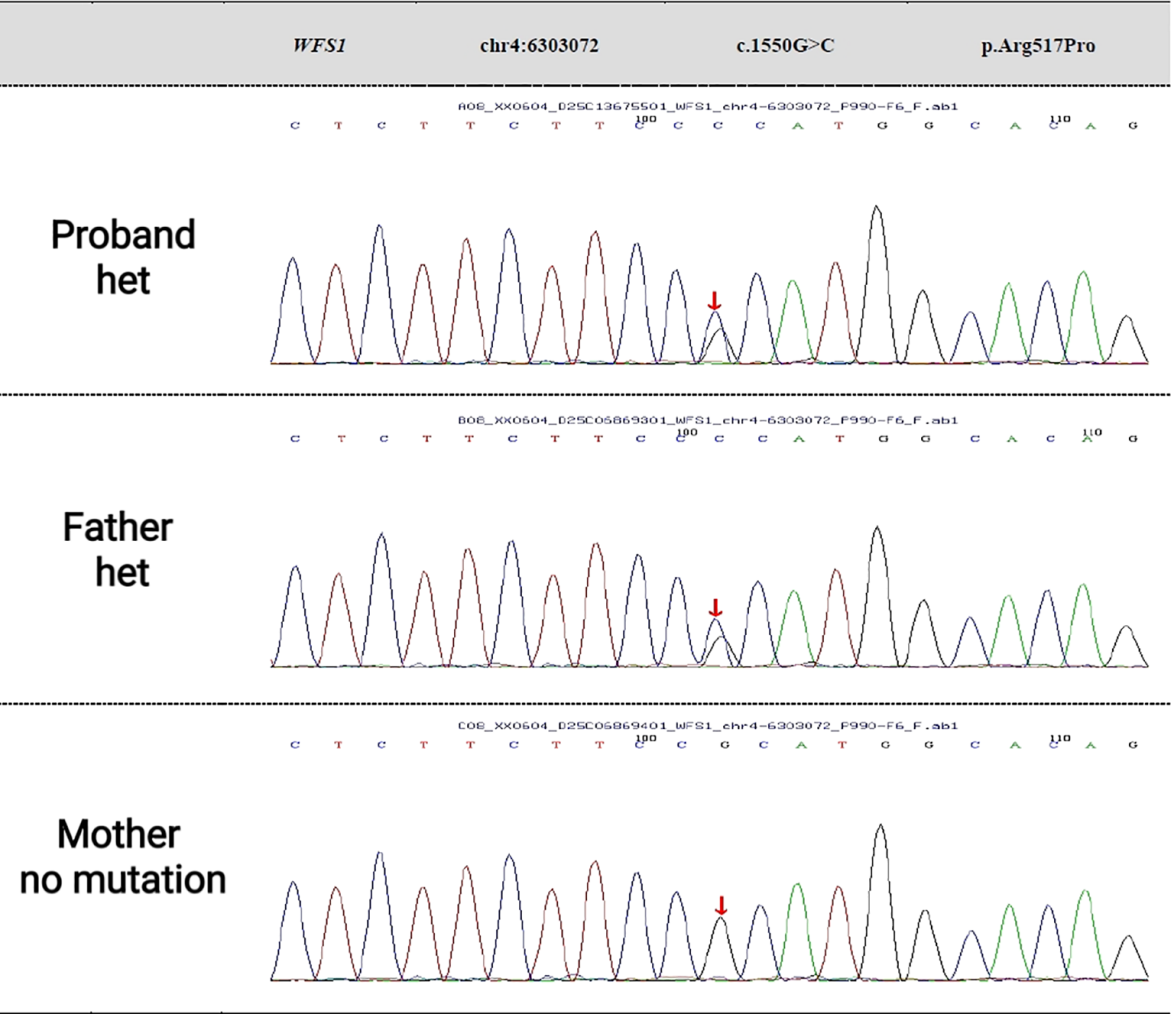
Gene sequencing results of the proband and his parents.

Peripheral venous blood (2 ml) was collected from the proband and their father and mother in February 2025. Whole-exome sequencing was conducted by KingWise with an average sequencing depth of 256.45× and a target region coverage of 99.78%. The pathogenicity of the variants was classified according to the ACMG guidelines. WES identified a heterozygous c.1550G>C (p.Arg517Pro) variant in the WFS1 gene. Based on the ACMG guidelines, this variant was classified as one of VUS. The classification was supported by the following criteria: PM2_Supporting (due to its extremely low allele frequency of 0.000077 in population databases) and PP3_Moderate (based on in silico predictions from the REVEL tool, which suggested a deleterious effect). No prior reports for this specific variant were found in the literature. Family analysis revealed that the patient’s father carries the heterozygous mutation at this locus, while the patient’s mother does not carry the mutation. (See **Figure 2** for details.).

### 2.5 Follow-up clinical indicators

The patient’s key clinical indicators and corresponding treatment regimens at baseline and during follow-up are summarized in **Table 1**.

**Table 1:** Clinical indicators and treatment regimens of the patient at baseline and follow-up.

Data	Treatment	Fasting blood glucose (mmol/L)	2-hour post prandial blood glucose (mmol/L)	Glycated hemoglobin (%)	Fasting C-peptide (ng/mL)
2023-02	Traditional Chinese medicine	11-12	–	–	–
2023-11	IdegAsp:12 U before breakfast and before dinner	11.17	–	9.3	1.16
2024-01	IdegAsp:12 U before breakfast and before dinner	6.45	7-8	–	0.98
2024-05	IdegLira 10 U before breakfast and IdegAsp 18 U before dinner combined with oral metformin (0.5 g t.i.d.) and chiglitazar sodium (32 mg q.d.).	6.24	6-7	5.6	0.79
2024-06	IdegLira 10 U before breakfast and IdegAsp 18 U before dinner combined with oral metformin (0.5 g t.i.d.) and chiglitazar sodium (32 mg q.d.).	5.03	5-7	–	0.79
2024-08	IdegLira 10 U before breakfast and IdegAsp 18 U before dinner combined with oral metformin (0.5 g t.i.d.) and chiglitazar sodium (32 mg q.d.).	4.64	–	5.7	0.76
2025-02	IdegLira 10 U before breakfast and IdegAsp 18 U before dinner combined with oral metformin (0.5 g t.i.d.) and chiglitazar sodium (32 mg q.d.).	7.66	–	6.7	0.96

IDegAsp, insulin degludec/aspart injection; IDegLira, insulin degludec/liraglutide injection; U, units; t.i.d., ter in die (three times daily); q.d., quaque die (once daily).

## 3 Discussion

WS typically presents with childhood-onset DM as its initial manifestation, occurring in approximately 98.21% of patients, while OA develops in about 82.14% (12). However, the phenotypic spectrum is heterogeneous, with only around half of the patients exhibiting the complete “DIDMOAD” tetrad of DM, OA, D, and DI. Over 200 pathogenic variants in the WFS1 gene have been associated with WS, which is commonly inherited in an autosomal recessive manner (13). Notably, some variants—particularly dominant missense mutations—are linked to milder or incomplete phenotypes. These can manifest as adult-onset DM, isolated symptoms, or the more broadly defined Wolfram-like disorder. Consequently, some scholars propose the term “WFS1-related diabetes” to encompass this phenotypic spectrum (2).

**Figure 3:**
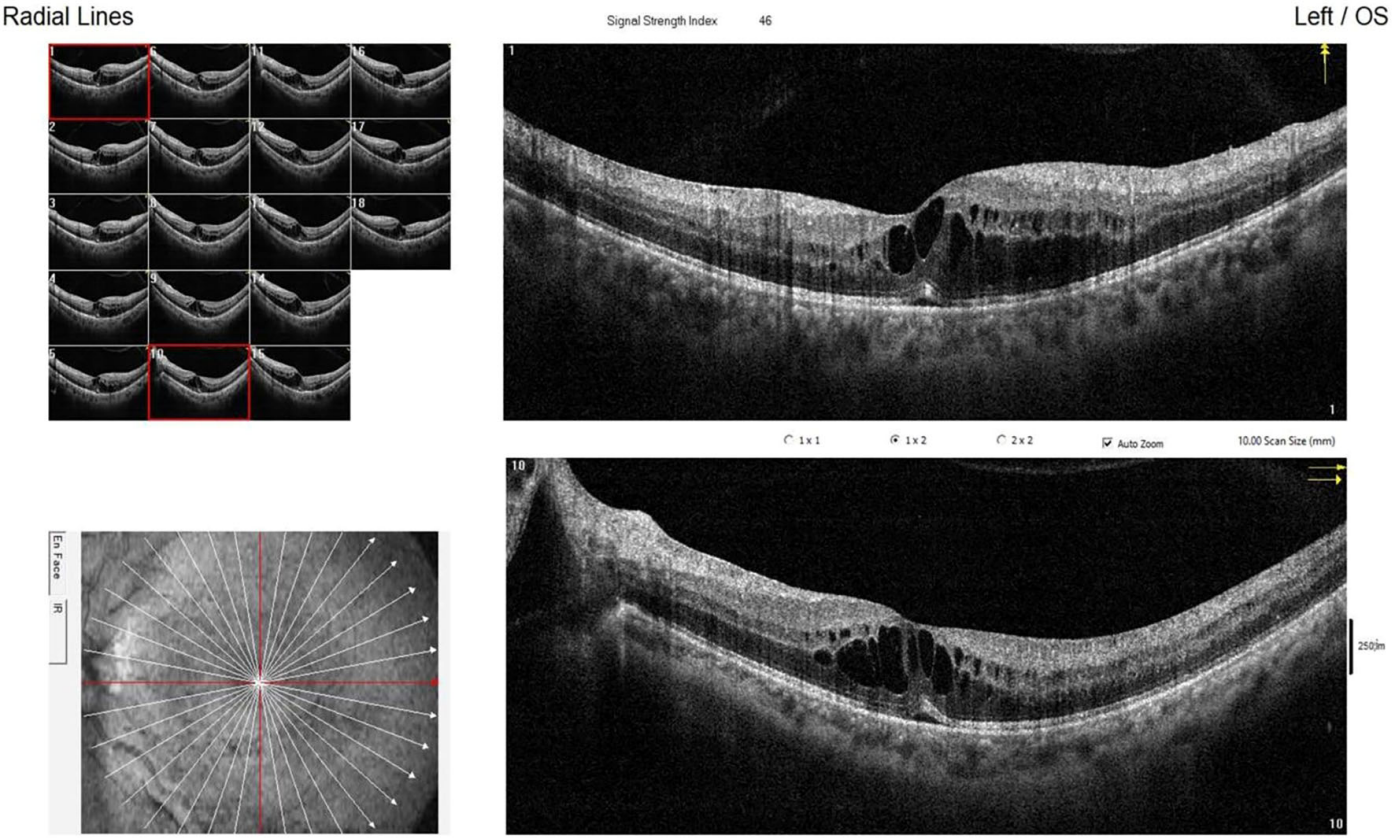
Optical Coherence Tomography.

**Figure 4:**
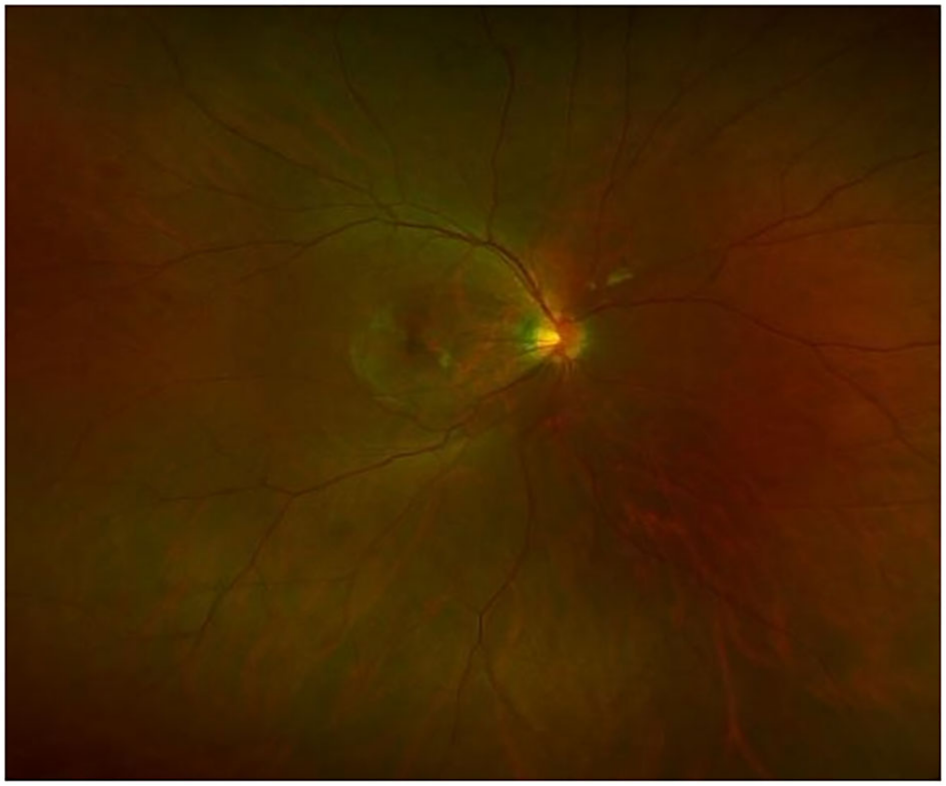
Laser Scanning Ophthalmoscopy.

The present case exhibits markedly atypical features: the age of DM onset was significantly delayed to 28 years, with an absence of typical neurosensory symptoms such as OA, deafness, or DI. No evidence of optic disc pallor and thinning of the retinal nerve fiber layer was found. (**Figures 3**, **4**) The patient’s clinical presentation was strictly confined to DM and its vascular complications (proliferative diabetic retinopathy with vitreous hemorrhage). Additionally, the patient’s grandfather, aunt, and uncle also exhibited only diabetes mellitus. A heterozygous WFS1 missense variant, c.1550G>C (p.Arg517Pro), was identified. This variant, located in exon 8—a region enriched for reported pathogenic missense changes (10, 14). It results in the substitution of a positively charged arginine residue at position 517 with proline. The pathogenic potential and mechanism of this heterozygous VUS remain speculative. Based on its location and radical amino acid change, one hypothetical mechanism could be a dominant-negative effect, where a mutant protein subunit disrupts the function of the wild-type protein complex, potentially leading to endoplasmic reticulum stress. However, this is purely conjectural in the absence of functional studies. To explore other genetic contributors, we performed a systematic re-analysis of the WES data. While variants in genes such as CRB1 and ACE were noted as potential modifiers of retinal and vascular risk, no other strong monogenic cause for the proband’s core phenotype was identified.

The familial segregation presents a critical puzzle. The proband’s father carries the same heterozygous p.Arg517Pro variant but is reported to be non-diabetic and asymptomatic. This is more like a phenomenon reported in certain WFS1 variants. An alternative explanation is that the variant is a benign rare polymorphism or a VUS with low or context-dependent pathogenicity. The multi-generational history of diabetes on the paternal side may reflect a coincidental aggregation of common diabetes, rather than segregation of the WFS1 variant.

In summary, this case report describes a novel WFS1 VUS (p.Arg517Pro) identified in an individual with an atypical diabetic phenotype lacking syndromic features. It underscores the complexity of interpreting rare heterozygous variants in genes associated with recessive disorders. While this finding contributes to the catalog of rare WFS1 variants encountered in clinical practice, it does not conclusively establish pathogenicity. The case highlights that WFS1 may be considered in the genetic evaluation of atypical, especially early-onset, diabetes, even in the absence of full syndromic features. For asymptomatic carriers of such VUS, prospective clinical monitoring may be prudent, though clear management guidelines await more definitive evidence of variant-specific risk. Future functional studies and reports of similar variants are needed to clarify the clinical significance of p.Arg517Pro and comparable WFS1 changes.

## 4 Limitations and future directions

This study has several important limitations. Most critically, the WFS1 p.Arg517Pro variant remains classified as a variant of uncertain significance (VUS). While its rarity and in silico predictions are notable, a definitive causal relationship with the patient’s phenotype has not been established. The interpretation presented here—including the possibility of a dominant-negative effect or incomplete penetrance—remains a speculative hypothesis requiring validation. Furthermore, the absence of detailed phenotypic data from the heterozygous father limits our ability to assess segregation and penetrance confidently.

To address these gaps and clarify the variant’s clinical significance, the following directions for future investigation are proposed:

1. Functional Studies: *In vitro* or cellular models (e.g., expressing the p.Arg517Pro variant) could assess its impact on wolframin function, endoplasmic reticulum stress, and potential dominant-negative interactions, providing direct mechanistic insight.
2. Extended Familial Analysis: Comprehensive phenotypic profiling (including audiologic, detailed ophthalmologic, and neurological evaluations) and genotyping of additional family members, starting with the father, would help delineate segregation patterns and better define penetrance.
3. Population Screening: Targeted screening for this variant in larger cohorts of patients with isolated early-onset diabetes or atypical Wolfram spectrum features could provide epidemiological data on its association strength and population frequency.
4. Comprehensive Genomic Interrogation: Techniques such as whole-genome sequencing could be employed to systematically identify potential cis- or trans-acting modifiers in non-coding regions or other genes that might contribute to the phenotype.

## 5 Conclusion

This case report describes the identification of a novel heterozygous WFS1 missense variant, c.1550G>C (p.Arg517Pro), currently classified as a variant of uncertain significance (VUS), in a family with a history of early-onset diabetes. The proband presented with adult-onset diabetes and severe vascular complications but lacked the characteristic neurosensory features of classical Wolfram syndrome. The variant was inherited from the father, who is reported to be a non-diabetic carrier, a finding that complicates its interpretation but is consistent with the phenomenon of incomplete penetrance reported for some WFS1 variants.

Integrating the clinical presentation, familial segregation, and the variant’s location within a functionally relevant domain, isolated diabetes may emerge as the predominant manifestation in this case. Consequently, the inclusion of WFS1 in the genetic evaluation of patients with atypical, especially familial early-onset, diabetes appears warranted, even in the absence of syndromic features. For asymptomatic individuals identified as carriers of similar VUS, clinical vigilance and consideration of periodic metabolic screening may be prudent, though definitive management guidelines await further evidence.

Ultimately, the pathogenic role and inheritance pattern of the p.Arg517Pro variant remain to be conclusively defined. Future functional studies and independent observations in other families are crucial to clarify its clinical significance. This report contributes a detailed phenotypic description to the growing catalog of rare WFS1 VUS and highlights the ongoing diagnostic and interpretive challenges within the Wolfram syndrome spectrum disorders.

## Data Availability

The original contributions presented in the study are included in the article/supplementary material. Further inquiries can be directed to the corresponding author.
